# Spiritually grounded character: A latent profile analysis

**DOI:** 10.3389/fpsyg.2022.1061416

**Published:** 2023-01-12

**Authors:** Tom Ford, Josh Lipson, Lisa Miller

**Affiliations:** Spirituality and Psychology Laboratory, Department of Clinical Psychology, Teachers College, Columbia University, New York, NY, United States

**Keywords:** spiritually grounded character, spirituality, character, positive psychology, latent profile analysis

## Abstract

**Introduction:**

The relationship between personal spirituality and character strengths has not been adequately explored. We investigate this relationship in an adult sample via latent profile analysis.

**Methods:**

Seven-hundred and sixty-three individuals aged 18 to 68years completed a survey of personal spirituality (e.g., Delaney Spirituality Scale), character strengths and virtues (e.g., VIA Inventory), flourishing (i.e., general wellbeing; PERMA Profiler), and various demographic variables (e.g., age, race, sexual orientation, yearly income, education level, religiosity, importance of spirituality and religion, and religious attendance). Latent profile analysis (LPA) was performed to identity profiles of individuals based on their reported levels personal spirituality and character strengths (i.e., VIA virtues).

**Results:**

A best-fitting model consisting of four distinct, non-overlapping profiles emerged. In every profile, the degree of personal spirituality was consistently associated with strengths of character. Profiles that exhibited high levels of spirituality and character also reported greater levels of flourishing. Furthermore, profiles high in spirituality and character were associated with the observance of formal religion, report of high levels of spiritual and religious importance, and report of frequent attendance of religious services.

**Discussion:**

These findings suggest that spirituality and character go hand in hand, that higher levels of the conjoint spirituality and character or “spiritually grounded character” correspond to greater wellbeing and may be generated or supported by a formal religious identification, practice, and community.

## Introduction

Spirituality and character development are closely related in many of the world’s cultures (Peterson and Seligman, 2004). From a reading of the major religious texts of the world, including the Ten Commandments, the Noble Eight-Fold Path, the Qur’an, the Eight Limbs of Yoga, and others, one readily observes how these major religious texts emphasize not only the principle of transcendence, but also principles of right behavior, thought, and speech. That these principles appear connected has long engaged philosophers. While philosophical speculations are beyond the scope of this paper, it behooves researchers to investigate the matter empirically. In the present paper, we ask: are character strengths and spirituality observably related? More specifically, are there latent profiles of individuals characterized by level of spirituality and character? Further, what are the psychological outcomes of those who have a rich spiritual life, high character, or both, compared to those who do not?

Spirituality, while hard to define, has been generally articulated by scientists as the search for, or communion with, the sacred (Pargament, 2013). The word “sacred” most commonly refers to God, a higher power, divinity, or qualities associated with the divine, such as transcendence, ultimacy, boundlessness, and a deep connectedness (Niemiec et al., 2020). People may experience the sacred through a variety of channels and practices, such as prayer, meditation, spiritual relationships, mystical experience, or felt connection to nature. The term “search” refers to a process of discovering, maintaining, and at times transforming a relationship with the sacred, and people may search for the sacred both within and outside traditional religious contexts (Pargament, 2013; Davis et al., 2015). While the specific content of spiritual belief, practice, and experience varies from culture to culture, all cultures have a concept of an ultimate, transcendent, or divine force (Peterson and Seligman, 2004; Portnoff et al., 2017).

A growing body of scientific research has found that spirituality plays an important role in psychological wellbeing (e.g., Pargament, 2002; Paloutzian and Park, 2013; Miller et al., 2014), physical health (e.g., Jim et al., 2015; Koenig, 2015), and in psychological adjustment to negative life experiences (Gall and Guirguis-Younger, 2013; Jones et al., 2016). Spirituality has also been linked to prosocial behavior (Bonner et al., 2003; Einolff, 2013).

Character strengths have also been shown to be cross-culturally universal (Peterson and Seligman, 2004; McGrath, 2015). The character strengths within the *VIA* classification have been confirmed in people across nations, cultures, and beliefs, including people living in some of remote cultures and places (Biswas-Diener, 2006; Park et al., 2006; McGrath, 2015). Character strengths are defined as positive personality traits that are core to identity, elicit positive outcomes (e.g., improved well-being, relationships, health, meaning, and achievement), and contribute to the collective good (Niemiec, 2018). Peterson and Seligman (2004) were the first to bring the study of character to modern psychology. They widely surveyed texts in subjects such as ethics, morality, virtue, theology, psychology, religion, philosophy, and related fields. The result of their investigation was the Values In Action (*VIA*) classification of strengths and virtues, a common language of 24 qualities that make us most and uniquely human. These 24 character strengths are then nested within six overarching virtue categories. For example, “creativity” and “love of learning” fall under the virtue of “wisdom,” while “bravery” and “honesty” under the virtue of courage. Likewise, “social intelligence” and “love” fall under the virtue of humanity, whereas “fairness,” and “teamwork” under the virtue of justice. Finally, “forgiveness” and “prudence” represent the virtue of temperance, and “hope” and “gratitude” fall under the virtue of transcendence. The complete classification is shown in Table 1.

**Table 1:** The *VIA* classification of strengths and virtues.

Character strengths and virtues					
Humanity	Courage	Justice	Temperance	Wisdom	Transcendence
Kindness Love Social intelligence	Bravery Honesty perseverance Zest	Fairness Leadership Teamwork	Forgiveness Humility Prudence Self-regulation	Creativity Curiosity Judgment Love of learning Perspective	Appreciation of beauty and excellence Gratitude Hope Humor Spirituality

Evidence for the validation of the *VIA* classification has been demonstrated in over 700 studies. However, few have formally examined the *VIA* character strengths and virtues and their relation to spirituality. Most recently, Niemiec et al. (2020) surveyed the literature and theorized that spirituality and character were synergistic. They assert that developing one’s character strengths is beneficial to one’s spiritual life and deepening one’s spiritual life may encourage the development of character. These authors articulate that spirituality and strengths of character are both lines of development toward human *wholeness*, by which they mean a “new way of being in the world that involves a life-affirming view of oneself and the world, a capacity to see and approach life with breadth and depth and the ability to organize the life journey into a cohesive whole” (Niemiec et al., 2020, page 1). The specific path toward wholeness may either be the *grounding path* – where one places emphasis on developing character strengths, or the *sanctified path*, in which the focus is spiritual development *per se*. Furthermore, in the same paper Niemiec et al. outline various ways in which spirituality and character strengths may be conceptually integrated: (1) as a single strength, where *spirituality* is demarcated as one of the 24 *VIA* character strengths; (2) as a grouping of the “spiritually oriented” strengths, noting that many of the character strengths in the *VIA* are embedded in the sacred literatures of the world’s major religious traditions; (3) as a single virtue, where one may define that spirituality is nested within the *VIA* virtue of *transcendence*; (4) as all virtues, in which one would argue that all six virtues derive from the world’s religious and wisdom traditions; (5) as a grouping of all 24 character strengths as reflecting spirituality, as one might argue that each character strength is derived from a spiritual principle; and (6) as a superordinate or master virtue, in which spirituality serves as a higher order virtue from which all others descend. Empirical research is required to determine which of the six is true.

To date there has been only one study to utilize person-centered analytic approaches to examine the relationship between spirituality and character strengths. Barton and Miller (2015) utilized latent class analysis to examine potential latent profiles of spirituality and several positive psychological constructs, including *gratitude, general sense of life optimism, grit,* and *individual sense of purpose and meaning in life*. These authors found that level of personal spirituality and level of positive psychology traits were found to overlap in 83% of adolescents and emerging adults and in 71% of older adults. A minority subgroup demonstrated low spirituality but high positive psychology traits and were labeled as “virtuous humanists.” Overall, the authors suggest that spirituality and positive psychology go hand in hand.

While this study was pioneering in utilizing person-centered, latent class analysis to the study of spirituality and positive psychology, it is limited in that it did not utilize the full *VIA* classification of strengths and virtues. The present study intends to fill this much needed gap in the literature.

Finally, we hypothesize that like Barton and Miller (2015), various latent profiles will emerge from the sample to show that character strengths and spirituality go hand in hand, and that profiles exhibiting higher levels of spirituality and character will also exhibit greater flourishing and well-being.

## Materials and methods

### Participants

We collected the data for this cross-sectional study using a web-based battery of psychological self-report measures related to spirituality, positive psychology, and clinical psychopathology developed at Teachers College, Columbia University. Seven hundred and sixty-three participants (*Mean age* = 38 years) from a broad range of racial, ethnic, religious, and cultural backgrounds in the United States participated in this study. Participants completed questionnaires *via* Amazon MTurk, a widely used web-based survey-response platform. Participants were paid $4.00 for their involvement in the study, and took 54 min to complete it, on average. All participants provided informed consent and were mindful of our safeguards to maintain the security and confidentiality of their de-identified data. All surveys were collected using Qualtrics. This study was approved by the Institutional Review Board and complied with all established human subject research practices laid out by the Belmont Report, etc.

### Demographics

Participants reported their age, sex, race, sexual orientation, religious orientation, personal importance of spirituality and religion, highest level of educational attainment, and estimated yearly income. Overall, the mean age was 38; 20.9% of participants were between 18 and 29; 41% were between the ages of 30 and 39; 21.7% of participants were between the ages of 40 and 49; and 16.4% of participants were fifty or older. The sample was 56% male, 44% female, <0.1% transgender, and <0.1% non-binary. With regard to racial identity, participants were 71 percent white (includes white Hispanics) 18% Black or African American, 10% Asian American, 2% mixed race and < 1% of participants identified as “other.” With respect to sexual orientation, 75.6% of participants identified as straight, 2.2% identified as gay/lesbian, 20.5% identified as bisexual, 0.7% identified as questioning, 0.9% preferred not to report, and 0.1% reported their sexual orientation as “other.” For educational attainment, 52% had an undergraduate degree (BA/BS), 22.9% had a graduate degree (MA/MS/PhD), 13.5% reported having “some college,” 6.5% reported having an associate’s degree (AA), 4.8% reported having a high school degree, and 0.3% reported having “some high school.” Regarding income, 31.4% reported an income in the 50–75 k range, 23.9% fell in the 30–50 k range,13.3 fell in the 100–200 k range, 14.1% earned between 15 and 30 k, 9.4% earned less than 15 k, and 1.1% earned over 200 k. With respect to religious beliefs, the sample was 5% atheist, 7.2% agnostic, 0.7% Buddhist, 54.7% Catholic, 1.3% Hindu, 2% Jewish, 0.4% Muslim, 4.2% spiritual but not religious, 0.9% other, `16% Protestant, 4.9% Christian (other), and 2.8 had no religious affiliation. Finally, with regard to personal importance of religion and spirituality, 14.7% said it was not important at all, 17.5 percent said it was slightly important, 33.2% said it was moderately important, and 34.6% reported that it was highly important.

### Measures

*Positive Psychological Virtues* were measured by the Values in Action Institute’s Character Strengths Survey. This survey is a 72-question self-assessment that helps clarify respondents’ particular strengths of character. Twenty-four lower-level character strengths (e.g., curiosity, creativity, bravery, prudence) load as subgroups into six higher-order virtue categories (e.g., courage, humanity, justice). Participants endorsed prompts (e.g., “I am an original thinker”) to varying degrees (e.g., 5 = “very much like me” to 1 - “very much unlike me”). For a listing of the full classification, see Appendix A.

*Spirituality* was examined *via* the Delaney Spirituality Scale (DSS). This scale measures the spiritual beliefs, intuitions, lifestyle choices, practices, and rituals, through a reliable and validated instrument (Delaney, 2005). Participants rated the extent to which they agreed or disagreed on a 6-point Likert scale along three dimensions (with higher scores indicating higher level of endorsement): *Self-Discovery*, *Spiritual Relationship*, and *Eco-Awareness. Self-Discovery* refers to the extent to which respondents engage in self-reflection and search for meaning and purpose. *Spiritual Relationship* refers to the extent to which respondents engage in meaningful connections with their Higher Power as well as fellow living beings with a deep respect and reverence for life. Finally, *Eco-Awareness* refers to respondents’ sense of a sacred connection with nature and Life as a whole. The Delaney Spirituality Scale has shown to have good content validity (*CI Index* = 0.94), robust reliability (Cronbach’s alpha =. 94 Delaney, 2005, and strong internal consistency (alpha = 0.916).

*Well-Being* was measured by the PERMA Profiler (Butler and Kern, 2016). The PERMA Profiler is a 23-item general measure developed for adults which assesses the five pillars of well-being as outlined in Martin Seligman’s theory of wellbeing (Seligman, 2012): positive emotion, engagement, relationships, meaning, and accomplishment. The measure demonstrates internal cross-time consistency, as well as evidence for content, convergent, and divergent validity (Butler and Kern, 2016).

### Descriptive statistics

First, we tabulated and processed the data using SPSS version 27. We verified the demographic profile of our participants and checked for the presence of any missing values. There were two missing values for the ‘highest level of education’ demographic, and four missing responses to the ‘estimated yearly income’ demographic. Besides these, there no other missing values in any other response domain from the 763 participating respondents. We subsequently computed correlation indices to understand the relationships between the various indicators. Table 2 summarizes these correlations.

**Table 2:** Pearson correlation among participant scores on the DSS and the *VIA*.

	Delaney Spirituality Scale	Humanity (*VIA*)	Courage (*VIA*)	Temperance (*VIA*)	Justice (*VIA*)	Wisdom (*VIA*)	Transcendence (*VIA*)
Delaney Spirituality Scale	–						
Humanity (*VIA*)	0.664**	–					
Courage (*VIA*)	0.628**	0.745**	–				
Temperance (*VIA*)	0.639**	0.651**	0.715**	–			
Justice (*VIA*)	0.607**	0.757**	0.692**	0.694**	–		
Wisdom (*VIA*)	0.631**	0.726**	0.783**	0.725**	0.720**	–	
Transcendence (*VIA*)	0.788**	0.768**	0.777**	0.741**	0.741**	0.768**	–

**Correlation is significant at the *p* < 0.01 level (2-tailed).

### Latent profile analysis

Latent profile analysis (LPA) is a model-based approach that offers several objective criteria that assist in determining goodness of fit and final model selection (Pastor et al., 2007), and it falls in the family of mixture modeling, latent variable approaches, and person-centered analytical techniques.

We used LPA to identify unique profiles of Spiritual Character based on their self-reported levels of positive psychological virtues as measured by the *VIA* and spirituality as measured by the DSS. More specifically, we based our analysis on the *VIA* virtue classes (e.g., Courage, Humanity, Justice, Temperance, Wisdom, and Spirituality) and DSS composite sum scores.

To conduct this LPA, we used the R package *tidyLPA* (v. 1.0.8 Rosenberg et al., 2018). This package allows scientists to derive mixture models and conduct cluster analysis through free open-source software. *TidyLPA* also allows researchers to control for variances and covariances among indicators in profiles. In addition, *tidyLPA* allows variances and covariances to vary freely across models and profile solutions. This provides maximum freedom to compare models and search for the most parsimonious solutions. A thorough overview of the various parameterization models in LPA may be found in Pastor et al., 2007.

Following the approach recommended by Pastor et al. (2007) and De Souza Marcovski and Miller (2022), we assessed the fit indices and model fit solutions from various parameterization approaches, allowing variances to vary or remain equal, and for covariances to vary or remain equal or be kept at zero across several models. Table 3 summarizes the various fit indices for each model across the various parameterizations we examined.

**Table 3:** Fit indices and LPA results with Models A, B, C, and D and their profile solutions indicating AIC, BIC, Entropy, and bootstrapped likelihood ratio test.

Model	Classes	AIC	BIC	Entropy	Min. probability	Smallest *n* (%)	BLRT (*p)*
Model A (equal variances; covariances fixed to zero)	1	15,178	15,243	1	1.00	1.00	-
	2	12,640	12,742	0.9	0.98	0.41	0.01
	3	11,631	11,770	0.89	0.95	0.21	0.01
	4	11,194	11,370	0.89	0.94	0.07	0.01
Model B (varying variances; covariances fixed to zero)	1	15,178	15,243	1	1	1	-
	2	12,433	12,567	0.91	0.97	0.46	0.01
	3	11,222	11,426	0.90	0.94	0.23	0.01
	**4**	**10,667**	**10,941**	**0.90**	**0.93**	**0.16**	**0.01**
Model C (equal variances; equal covariances)	1	10,466	10,628	1	1	1	-
	2	10,308	10,507	0.90	0.85	0.11	0.01
	3	10,324	10,561	0.43	0.16	0.1	0.96
	4	10,319	10,593	0.49	0.25	0.07	0.49
Model D (varying variances; varying covariances)	1	10,466	10,628	1	1	1	-
	2	9,638	9,968	0.72	0.84	0.31	0.01
	3	9,525	10,021	0.70	0.83	0.21	0.01
	4	9,495	10,158	0.74	0.77	0.07	0.08

AIC, Akaike Information Criterion; BIC, Bayesian Information Criterion; BLRT, Bootstrapped likelihood ratio test; Min. Probability: the minimum probability value assignment to one of the classes within the profile solution. Boldened row indicates the selected solution. Number of profile solutions (classes) range from one to five.

As noted, *tidyLPA* allows for four specific types of parameterization. Model 1 is the most constrained and restrictive model, with variances are held equal across profiles and covariances between indicators are fixed to zero. Model 2 offers more flexibility, as variances are allowed to freely vary across profiles, while covariances remain fixed to zero. Model 3 allows greater flexibility, in that variances are allowed to vary freely across profiles but allows the covariances among the six indicators to be estimated and held equal across the profiles. Finally, model 4 provides the greatest flexibility, where both variances and covariances are allowed to freely vary between and within profiles.

To assess model fit solutions, we recognized the Akaike Information Criterion (AIC; Akaike, 1987), the Bayesian Information Criterion (BIC; Schwarz, 1978), and entropy as the main indices. We prioritized parsimonious solutions and performed visual inspections of every profile solution to select a final model, while considering confidence intervals and statistically significant differences between indicators.

### Analysis of variance

Finally, to examine mean differences in wellbeing between the profiles of Spiritual Character, we used one-way analysis of variance (ANOVA). Like previous studies in this area of inquiry (Barton and Miller, 2015), we used participants’ most likely profile membership as our independent factor to predict differences in the dependent factor, wellbeing. In addition, we conducted cross-tabulations and chi-square tests of proportions to identify any significant differences in demographic characteristics (e.g., religious identity and degree of religious and spiritual importance) between the profiles. When conducting multiple comparisons, we used Tukey’s HSD to control for Type I errors.

## Results

Our first aim was to assess the relationships within the six virtues of character of the *VIA* (e.g., Courage, Humanity, Justice, Temperance, Wisdom, and Transcendence), as well as with the Delaney Spirituality Scale (DSS) (Table 2). Among the character virtues, we found significant medium to strong intercorrelations. These intercorrelations have been documented in previous studies, although these correlations appear to be stronger in the present analysis than in those past (Ruch and Proyer, 2015; Giuliani et al., 2020). Every virtue was highly correlated with the spirituality as measured by Delaney (ranging from *r* = 0.63–0.79), showing significant medium-to-strong associations.

Our second aim was to identify profiles of individuals among the sample using the spirituality scale and the six *VIA* character virtues as indicators. Table 3 displays results from latent profile analyses (LPA), including indices of model fit such as Akaike Information Criterion (AIC), Bayesian Information Criterion (BIC), level of entropy, and bootstrapped likelihood ratio test *p*-values for each profile of participants within every model generated by the *tidyLPA* software. We programmed the software to record models from one and four profile solutions because models with more profiles might potentially detract from explanatory clarity as well as reduce the numbers of participants in each profile, thus posing a threat to statistical power and generalizability. Lower values for AIC and BIC suggest better-fitting models, and higher levels of entropy indicate greater classification accuracy and precision of the model.

**Figure 1:**
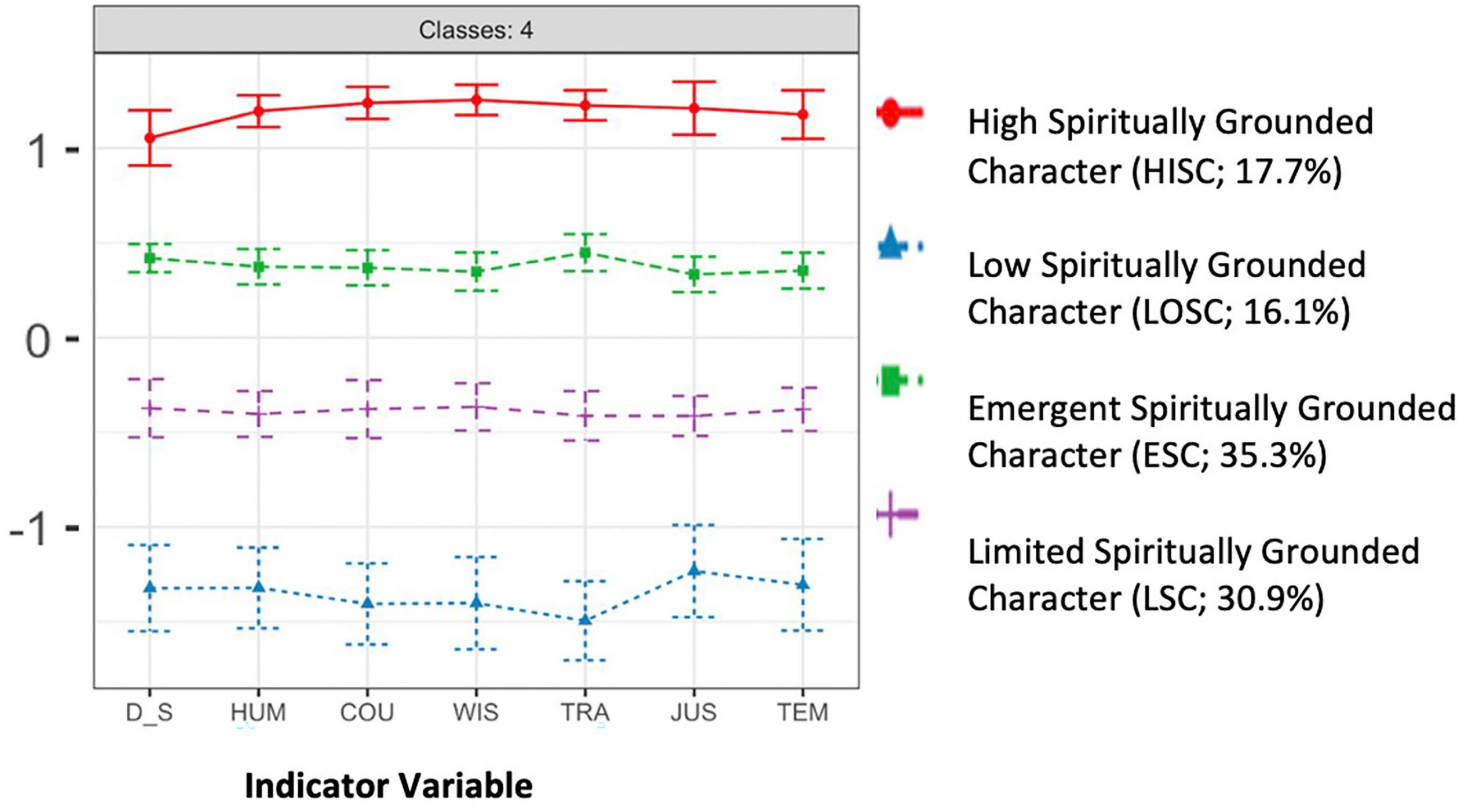
Line graph indicating the four-class solution featuring standardized mean values on the *y*-axis and the indicators used on the x-axis. *D_S*, Delaney Spirituality; HUM, Humanity; COU, Courage; WIS, Wisdom; TEM, Temperance; TRA, Transcendence; JUS, Justice. Bars represent 95% Confidence Intervals around the standardized value for a given indicator. Profile 1: High Spiritually Grounded Character (Mean Age 19%); Profile 2: Evident Spiritually Grounded Character 2: (17%); Profile 3: Limited Spiritually Grounded Character (36%); Profile 4: Low Spiritually Grounded Character (28%).

As our results indicate, Model D (which allows for both variances and covariances to be freely estimated and varied across profiles) featured the lowest, and therefore best-fitting AIC and BIC values. However, this complex parameterization came at the expense of confidence of precision in the classification of participants into the correct profile (i.e., compared to other models, entropy in Model D was relatively low, between 0.7 and 0.83). The next lowest AIC and BIC values were featured in Model C, which specified parameters of equal variances and equal covariances) However, this model was also discarded because either entropy levels were either too low, threatening the precision of participant classification to profile (i.e., 0.49 or 0.43), or the number of generated profiles (e.g., 2) held little explanatory or conceptual significance. Thus, we settled on the four-class solution for Model B, which allowed variances to vary while fixing covariances to zero. This model had relatively low AIC and BIC values while also showing a very high entropy value, suggesting precision in the classification participants to the correct profile. Furthermore, the minimum probability was high (0.93), indicating classification accuracy and the bootstrapped likelihood ratio test (BLRT) was low (0.01), indicating goodness of model fit. Finally, this four-profile solution also provided the greatest explanatory power on the basis of visual inspection. That is, while earlier studies in the literature provide three class models showing “high,” “medium” and “low” profile solutions, the present study adds greater complexity and specificity by including an additional profile to include high, high-medium, low-medium, and low solutions. The four profiles were labeled in terms of their degree of spiritually integrated character: the profile demonstrating the greatest values across the indicators was labeled “High *Spiritually Grounded Character*” (Hi*SGC*), the medium-high profile was labeled “*Evident Spiritually Grounded Character*” (*ESGC*), medium-low group was labeled “*Limited Spiritually Grounded Character*” (*LSGC*), and finally, the lowest group was labeled “*Low Spiritually Grounded Character*” (*LoSGC*). Figure 1 illustrates this model solution.

Table 4 summarizes the standardized mean scores and standard deviations of each of the indicator variables among the four profiles of the model. Higher scores indicate higher levels of each indicator variable (e.g., Delaney Spirituality, Humanity, Courage, Justice, Wisdom, Temperance, and Transcendence). Consistent with earlier studies (e.g., Barton and Miller, 2015), spirituality as measured by the DSS goes hand in hand with character virtues outlined by Peterson and Selgiman (2004). As our results show, 17.7% (*n* = 135) of the sample was classified to the *High Spiritually Grounded Character* (HiSGC; mean age = 37) profile (*z*-scores ranging from 1.06 to 1.26), 35.3% (*n* = 269) to the *Evident Spiritually Grounded Character* (ESGC; mean age = 39) profile (*z*-scores ranging from 0.33 to 0.45), 30.9% (*n* = 236) to the *Limited Spiritually Grounded Character* (LSGC; mean age = 39) profile (*z*-scores ranging from −0.38 to −0.43), and 16.1% (*n* = 123) to the *Low Spiritually Grounded Character* (LoSGC; mean age = 36) profile (z-scores ranging from −1.51 to −1.25). In our one-way ANOVA, each of these profiles was significant at the *p* < 0.001 level, indicating that they represent meaningful and distinct classifications. Of note, the more extreme profiles showed half the representation as the moderate profiles, potentially suggesting a normal distribution of Spiritually Grounded Character.

**Table 4:** Standardized mean scores and standard deviations of indicator variables in each profile.

	High spiritually grounded character (17.7%)	Emergent spiritually grounded character (35.3%)	Limited spiritually grounded character (30.9%)	Low spiritually grounded character (16.1%)
	*M* (SD)	*M* (SD)	*M* (SD)	*M* (SD)
Del. Spirituality	1.06 (0.53)	0.42 (0.45)	−0.39 (0.65)	−1.33 (0.99)
Humanity	1.2 (0.39)	0.38 (0.47)	−0.42 (0.62)	−1.33 (0.92)
Courage	1.24 (0.4)	0.37 (0.47)	−0.38 (0.57)	−1.45 (0.72)
Wisdom	1.26 (0.37)	0.35 (0.45)	−0.36 (0.54)	−1.45 (0.82)
Temperance	1.18 (0.57)	0.35 (0.53)	−0.38 (0.6)	−1.32 (0.88)
Justice	1.22 (0.53)	0.33 (0.5)	−0.42 (0.59)	−1.25 (0.95)
Transcendence	1.23 (0.37)	0.45 (0.4)	−0.43 (0.44)	−1.51 (0.74)

The third aim of our study was to determine whether the profiles of spiritual character identified in the model differed with respect to measures of general wellbeing *via* the PERMA Profiler and its five pillars and respective subscales (i.e., positive emotion, engagement, relationships, meaning, and accomplishment). To accomplish this, a one-way ANOVA was conducted that used profile membership (e.g., 1, 2, 3, or 4) as the independent categorical variable, and the PERMA profile subscales as the dependent variables. Significant differences in each wellbeing domain were found between every profile of spiritual character (*p* < 0.001). Table 5 summarizes the means, standard deviation, and *F* statistics comparing the reported means across profiles. Of note, because all mean scores were derived from a single wellbeing scale, there was no need to use standard scores.

**Table 5:** Means and standard deviations of PERMA Profiler scores by profile membership; *F* statistic of one-way ANOVA tests.

	High spiritually grounded character	Emergent spiritual character	Limited spiritual character	Low spiritual character	*F*	Sig
	*M (*SD)	*M (*SD)	*M (*SD)	*M (*SD)		
PERMA flourishing overall	153.6 (16.6)	139.78 (19.14)	120.19 (21.26)	92.04 (27.29)	227.76	0.000
Positive emotion	29.26 (3.74)	26.12 (4.2)	21.94 (5.05)	16.25 (6.55)	188.26	0.000
Engagement	27.27 (3.95)	25.25 (4.43)	22.35 (3.98)	18.15 (5.78)	109.75	0.000
Relationships	28.94 (4.47)	26.86 (4.43)	22.89 (5.78)	17.44 (6.82)	128.54	0.000
Meaning	29.36 (3.72)	26.67 (4.19)	22.75 (5.19)	17.58 (6.93)	149.37	0.000
Accomplishment	28.91 (3.64)	26 (4.05)	22.62 (4.48)	17.34 (5.66)	175.05	0.000

The *HiSGC* profile was associated with the highest levels of every domain of wellbeing, followed by the *ESGC, LSGC, and LoSGC* profiles, in respective order. The HiSGC profile overall wellbeing mean score was 153.6, and subscale scores ranged between 27.27 and 29.36. The ESGC profile overall wellbeing mean score was 139.78 with subscale mean scores ranging from 25.25 to 26.86. The LSGC profile overall wellbeing mean score was 120.19 with subscale mean scores ranging from 21.94 to 22.89. Finally, the LoSGC profile overall wellbeing mean score was 92.04 with subscale mean scores ranging from 16.25 to 18.15.

Finally, we assessed for any differences in religious adherence among the four profiles, namely in: identification with religious traditions, degree of religious/spiritual importance and frequent attendance of religious services. Individuals classified to the High Spiritually Grounded Character profile were the most likely to identify with a particular religious tradition (91%; *n* = 123), report religion or spirituality as “highly important (55%; *n* = 74), and attend weekly religious services (50%; *n* = 68). The Evident Spiritually Grounded Character profile was the second most likely to identify with a religious tradition (89%; *n* = 239), the second most likely to endorse religion and spirituality as “highly important” (43%, *n* = 117), and the second most likely to attend weekly religious services (38%; *n* = 103). The Limited Spiritually Grounded Character profile was third most likely to identify with a religious tradition (76%; *n* = 180), endorse religion and spirituality as “highly important” (25%; *n* = 58), and attend weekly religious services (27%; *n* = 63); and finally, the Low Spiritually Grounded Character group was least likely to identify with a religious tradition (61%; *n* = 75), endorse religion and spirituality as “highly important” (12%; *n* = 15), and attend weekly religious services (13%, *n* = 16). Chi square tests of independence showed that there were significant differences between profiles for religious identification (*λ* = 79.66; df = 33; *p* < 0.001), religious and spiritual importance (*λ* = 104.2; df = 9; *p* < 0.001), and frequency of religious attendance (*λ* = 78.44; df = 12; *p* < 0.001).

## Discussion

The aims of the present study were: (1) to investigate the relationship between spirituality and character strengths, and (2) whether these variables were associated with human flourishing. To address these aims, we conducted a series of latent profile analysis, a person-centered cluster analytic approach using personal spirituality (i.e., Delaney Spirituality Scale Sum score) and the character virtues from the *VIA* inventory as indicator variables. We identified a four-profile solution which demonstrates that spirituality and character virtues go hand in hand, that is, the degree to which one exhibits personal spirituality is closely associated with their level of character (e.g., high, medium-high, medium-low, or low). We named these profiles as follows: *High Spiritually Grounded Character, Emergent Spiritually Grounded Character, Limited Spiritually Grounded Character,* and *Low Spiritually Grounded Character.* The profiles appear visually (see Figure 1) as four parallel horizontal lines, such that the level of spirituality is always matched with the level of each virtue. This suggests that there may be foundational links between spirituality and strength of character. Furthermore, profiles with higher levels of spiritually grounded character exhibited greater flourishing.

As noted in the introduction, there has been historically limited but increasingly growing interest in the relationship between spirituality and character strengths (e.g., Niemiec et al., 2020). To date only one study has applied similar person-centered approaches to the subject. Barton and Miller (2015) used latent class analysis to identify four profiles, three of which showed personal spirituality to be consistent with the positive psychology traits of grit, optimism, gratitude, forgiveness, and meaning in life. The fourth profile was called the “virtuous humanists,” who exhibited relatively low spirituality but elevated positive psychology traits.

The present study largely replicated the results from Barton and Miller (2015), with the notable addition of including all dimensions across the entire *VIA*, the full measurement of which stands as a widely shared standard for character strengths in the field of positive psychology.

The primary difference in the pattern of the findings between the current study and that of Barton and Miller, is that beyond three “parallel” levels (High, Medium, and Low) of spiritually grounded character found by Barton & Miller, the previous team also identified a single profile that appeared low in spirituality but high in positive psychological variables (i.e., the “virtuous humanists”), whereas the present study found no such group. Rather, in the current study all four profiles demonstrated consistent levels across personal spirituality and positive psychological variables (i.e., the *VIA* character virtues). This discrepancy in findings points to the value of further research. Researchers should continue to investigate whether level of personal spirituality is consistently in line with level of character, or whether there are some profiles for which the two variables are divergent.

The second difference between the two studies is in the age of the study sample. Barton and Miller looked exclusively at college age participants (18–24 years of age), whereas the current data set included people across other decades of life. Differences in the findings therefore may suggest that: (1) perhaps in late adolescence and emerging adulthood there is a developmental process of reconciling personal spirituality and character, and (2) across the decades of adult development the spirituality and character ultimately drawn into a common level.

The present findings further support the idea that character and spirituality are inextricably linked. However, the study does not show us *how* they are related. As yet, scientists can only theorize how the two may be related. Some may argue that spirituality is a higher-order virtue that influences all other virtues in the hierarchy. Others may assert that the virtues in the *VIA* are simply component parts of spirituality. The present study, however, suggests that personal spirituality and strength of character are connected, such that high spirituality is linked to high character, medium spiritual character is linked to a medium level of character, and a low level of spirituality is linked to a low level of character.

Many have theorized why spirituality and character are connected. One plausible explanation states that the degree to which one understands life and everything in it as *sacred* (Pargament, 2013) is the degree to which they to develop a general attitude of reverence, awe, and good will toward all things. Researchers would do well to continue this line of investigation on the “sanctification of daily life,” not least because of its implications for heathy citizenry. Additionally, scientists may embrace those expanded definitions of personal spirituality that include reference to the embodied relational moral or the ethical dimensions, or “walking the walk” of spiritual values, as opposed exclusively the transcendent ones. Relational spirituality has been understood by Sandage and colleagues (Sandage and Shults, 2007; Tomlinson et al., 2016), as both a relationship with a Higher Power as well as the perceived presence of the Higher Power in fellow human beings. Indeed, Miller et al. (2021) found a substantial overlap of the neural correlates associated with the two forms of relational spirituality, suggesting a potential overlap is spiritual perception.

It is worth noting in this study that those highest in both character and spirituality also tended to have a formal religious identity and to consistently attend religious services. There were certainly cases where individuals in the high spiritual character profile endorsed no religious identification and attended no services, and there were cases in the low spiritual character profile that endorsed a high level of religious importance, claimed a formal religious identity, and attended regular services. However, in each of these, cases were rare. We see that those with formal, ongoing religion tend to have higher levels of spiritual character. Many conclusions may be taken from this, including the possible civic and social benefits of engagement with the practice of religion, as well as the potential tenuousness of the popular “spiritual but not religious” identity toward character and thriving. These findings suggest that spirituality and character may be greatly supported by a formal religious engagement, formation of a religious identity, active religious practice, and strong religious community.

Finally, we see that spiritual character is highly connected to overall flourishing. The present findings show a uniform order of those who showing higher spiritual character also demonstrating greater flourishing. Here we see potential limitations of purely secular (or at least non-spiritual), positive psychological approaches to wellbeing. While these approaches are popular and effective, it appears that *in vivo* spiritual character is a potent force behind human flourishing and wellbeing. Scholars and practitioners interested in wellbeing may wish to make spirituality foundational to their work.

### Limitations and recommendations for future research

There are several limitations to this study. The first is that it utilizes cross-sectional data. This means that there can be no inference of causality. We cannot see from this study whether personal spirituality creates high character, or whether high character encourages spirituality, or whether actually the two dimensions foundationally are one, inextricable as one entity. We similarly cannot state whether the profiles cause high levels of flourishing. These findings can only suggest that there appear to be latent profiles of spiritually grounded character in the population showing a close connection between personal spirituality and character, and that profiles characterized by high spirituality and character strengths tend to be associated with high levels of flourishing.

Authors interested in the relation between spirituality and character must continue to investigate whether spirituality is a superordinate virtue that encompasses all others, character is deeper and more longstanding when rooted in spiritual awareness, or whether spirituality and the lived character virtues should be conceptualized as one entity or as distinct. This will require philosophical, theoretical, and empirical efforts. Scholars should continue to wrestle with definitions of spirituality and character on the philosophical-theoretical levels and may wish to employ techniques such as multilevel modeling (Peugh, 2010). The implications of this line of investigation are far reaching, and it may shift how we understand and encourage character development and general wellbeing.

Additionally, while the authors of the current paper used *tidyLPA* to conduct a latent profile analysis, there are other approaches to derive profiles, including hierarchical cluster analysis, bi-factor models, and exploratory structural equation modeling. Naturally we acknowledge that latent profile analysis is just one of several ways to identify profiles or clusters in a population, and we encourage authors to explore other methods to bolster the field.

Finally, these data were collected from a largely North American sample. We acknowledge this limitation, as it falls short of ideal, worldwide diversity of nationality, ethnicity, race, gender, and sexual orientation.

## Summary

The current study identified four distinct, non-overlapping latent profiles of spiritually grounded character using latent profile analysis (LPA). Each profile showed that personal spirituality and character virtues are integrated, that is, the level of personal spirituality consistently matched the level of character virtue. Profiles of higher spiritually grounded character consistently exhibited greater general wellbeing and flourishing. Finally, members of profiles showing higher levels of spiritual character also tended to be more formally religious, report higher levels of religious/spiritual importance, and higher rates of religious attendance.

Finally, theorists of spirituality and positive psychology might consider of spirituality as a superordinate-level virtue that encompasses every other virtue and strength of character. They may also explore ideas that character itself is an expression of embodied relational spirituality, or that or that it emanates from the perception or experience of relational spirituality. Spiritually grounded character may be used to increase the efficacy and depth of positive psychology interventions in adults and children, and it may be used to support whole-child education.

## Data availability statement

The raw data supporting the conclusions of this article will be made available by the authors, without undue reservation.

## Ethics statement

The studies involving human participants were reviewed and approved by Teachers College Institutional Review Board. The patients/participants provided their written informed consent to participate in this study.

## Author contributions

TF wrote every section and conducted analysis and interpretation. JL contributed to data collection, study administration, and idea generation. LM lead author and editor. All authors contributed to the article and approved the submitted version.

## Conflict of interest

The authors declare that the research was conducted in the absence of any commercial or financial relationships that could be construed as a potential conflict of interest.

## Publisher’s note

All claims expressed in this article are solely those of the authors and do not necessarily represent those of their affiliated organizations, or those of the publisher, the editors and the reviewers. Any product that may be evaluated in this article, or claim that may be made by its manufacturer, is not guaranteed or endorsed by the publisher.
